# miR-17-92a-1 cluster host gene: a key regulator in colorectal cancer development and progression

**DOI:** 10.1007/s10238-024-01331-1

**Published:** 2024-04-25

**Authors:** Amirhossein Mohajeri Khorasani, Samane Mohammadi, Alireza Raghibi, Behzad Haj Mohammad Hassani, Behina Bazghandi, Pegah Mousavi

**Affiliations:** 1 Department of Medical Genetics, Faculty of Medicine, Hormozgan University of Medical Sciences, Bandar Abbas, Iran; 2 Molecular Medicine Research Center, Hormozgan Health Institute, Hormozgan University of Medical Sciences, Bandar Abbas, Iran; 3 Student Research Committee, Hormozgan University of Medical Sciences, Bandar Abbas, Iran; 4 Department of Medical Genetics, School of Medicine, Tehran University of Medical Sciences, Tehran, Iran; 5 Protein Research Center, Shahid Beheshti University, Tehran, Iran

**Keywords:** MIR17HG, miR-17-92a-1 cluster host gene, Colorectal cancer, Non-coding RNA, miRNA

## Abstract

Colorectal cancer (CRC), recognized among the five most prevalent malignancies and most deadly cancers, manifests multifactorial influences stemming from environmental exposures, dietary patterns, age, and genetic predisposition. Although substantial progress has been made in comprehending the etiology of CRC, the precise genetic components driving its pathogenesis remain incompletely elucidated. Within the expansive repertoire of non-coding RNAs, particular focus has centered on the miR-17-92a-1 cluster host gene (MIR17HG) and its associated miRNAs, which actively participate in diverse cellular processes and frequently exhibit heightened expression in various solid tumors, notably CRC. Therefore, the primary objective of this research is to undertake an extensive inquiry into the regulatory mechanisms, structural features, functional attributes, and potential diagnostic and therapeutic implications associated with this cluster in CRC. Furthermore, the intricate interplay between this cluster and the development and progression of CRC will be explored. Our findings underscore the upregulation of the miR-17-92a-1 cluster host gene (MIR17HG) and its associated miRNAs in CRC compared to normal tissues, thus implying their profound involvement in the progression of CRC. Collectively, these molecules are implicated in critical oncogenic processes, encompassing metastatic activity, regulation of apoptotic pathways, cellular proliferation, and drug resistance. Consequently, these findings shed illuminating insights into the potential of MIR17HG and its associated miRNAs as promising targets for therapeutic interventions in the management of CRC.

**Graphical abstract:**

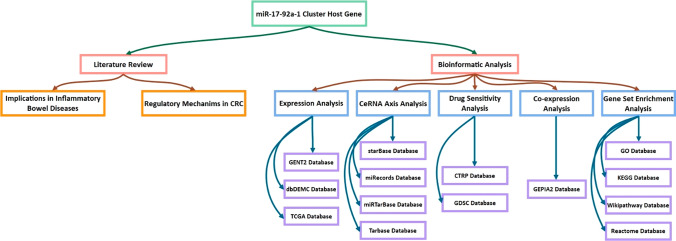

**Supplementary Information:**

The online version contains supplementary material available at 10.1007/s10238-024-01331-1.

## Introduction

Colorectal cancer (CRC) stands as a highly prevalent global affliction, ranking as the third most common cancer worldwide [1]. In terms of mortality, CRC assumes the grim position of being the second most lethal malignancy [1, 2]. Its incidence is markedly impacted by an interplay of environmental factors, lifestyle choices, dietary habits, age, and genetic predispositions [3]. While the full etiology of this disease remains elusive, substantial evidence has substantiated the pivotal role of genetic factors in its pathogenesis [4, 5].

Exceeding 75% of the human genome is subject to transcription; however, the majority of these transcripts do not undergo protein translation and are classified as non-coding RNAs [6]. Prominent categories of non-coding RNAs, including microRNAs (miRNAs), long non-coding RNAs (lncRNAs), and circular RNAs (circRNAs) [6, 7] have been elucidated for their pivotal roles in a multitude of diseases, especially diverse types of cancer [8]. These RNA species exhibit distinct attributes in terms of length, structure, and functionality. For instance, miRNAs typically comprise approximately 22 nucleotides, whereas lncRNAs are predominantly longer than 200 nucleotides [9]. Additionally, in recent years, the utilization of non-coding RNAs as non-invasive diagnostic biomarkers has gained significant popularity [10, 11]. Hundreds of microRNA clusters have been recognized within the human genome, including the miR17-92a-1 cluster host gene, also known as MIR17HG [12]. The miR-17–92 cluster host gene, located on human chromosome 13q31.3, is a gene cluster with a polycistronic promoter that gives rise to mature miRNAs: hsa-miR-17, hsa-miR-18a, hsa-miR-19a, hsa-miR-19b-1, hsa-miR-20a, and hsa-miR-92a. This cluster contributes to the development of malignancies and has considerable overexpression in a variety of solid tumors [13]. Mogilyansky et al*.*, in their study, show that the miR-18a and miR-20a are overexpressed in CRC [14]. Alternatively, XU et al. explored MIR17HG as a pivotal BLNK inhibitor, revealing that BLNK suppression enhances metastatic potential and tumorigenesis in CRC [15]. Additionally, Cellura and colleagues demonstrated that miR-19, through its inhibitory effects on transglutaminase 2, fosters augmented invasion and metastasis in CRC [16]. However, the generalizability of many published papers on this issue is limited, and such expositions are far from satisfactory due to their failure to undertake a thorough exploration into the regulatory mechanism, structure, function, and potential therapeutic implications of this specific cluster in CRC. Therefore, this study aims to comprehensively investigate the miR-17-92a-1 cluster host gene and its intricate regulatory relationship with the development and progression of CRC.

### miR-17-92a-1 cluster host gene overview

The miR-17-92a-1 cluster host gene (MIR17HG) is situated at 13q31.3, position 91,347,820 to 91,354,575, within the genomic sequence with accession number NC_000013.11 (GRCh38.p14 primary assembly). This gene has two linear long non-coding RNA variants called MIR17HG transcript variants 1 and 2, which have lengths of 5,018 bp (NR_027350.1) and 927 bp (NR_027349.1), respectively. MIR17HG, on the other hand, serves as a host gene for six microRNAs (hsa-miR-17, hsa-miR-18a, hsa-miR-19a, hsa-miR-19b-1, hsa-miR-20a, and hsa-miR-92a-1) (https://www.ncbi.nlm.nih.gov/gene/407975). Based on the NCBI database, we have added more information regarding the MIR17HG-derived RNAs as follows:

### MIR17HG LncRNA

**Fig. 1:**
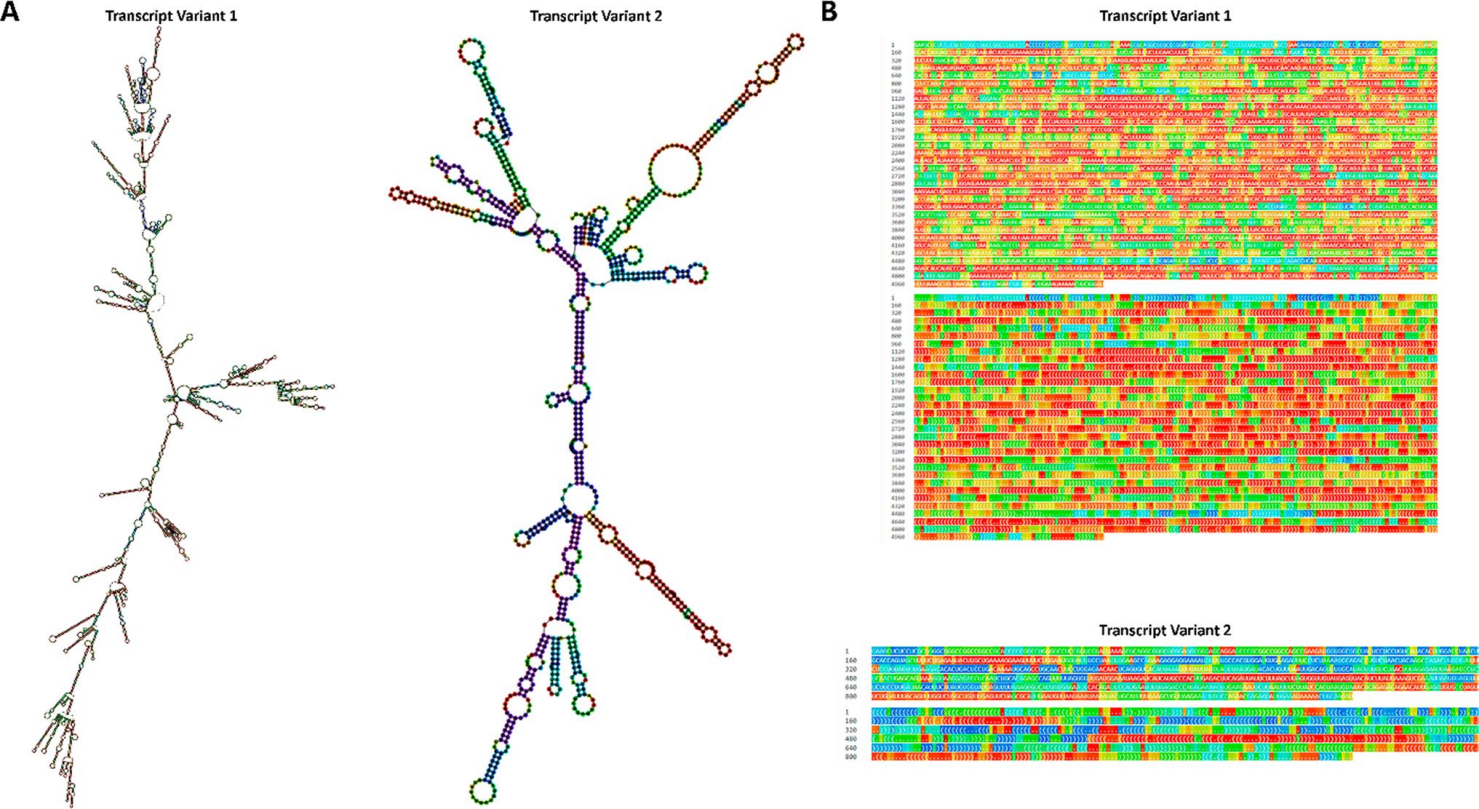
The optimal minimum free energy (MFE) construction **A** and dot-bracket representation **B** related to two ncRNA-derived MIR17HG transcript variants. The results showed that MIR17HG transcript variant 1 had a MFE of 1398.30 kcal/mol, while MIR17HG transcript variant 2 had a MFE of − 262.50 kcal/mol

The optimal secondary structure of transcript variants 1 and 2 for the non-coding RNA MIR17HG was analyzed using the RNAfold web server (http://rna.tbi.univie.ac.at/cgi-bin/RNAWebSuite/RNAfold.cgi) [17, 18]. The structures were drawn in both minimum free energy (MFE) and dot-bracket notation. The MFE structure for variant 1 was calculated to be -1581.60 kcal/mol, whereas that for variant 2 was -262.50 kcal/mol. The results suggest that the secondary structure of the MIR17HG transcript variants 1 and 2 is highly stable with low MFE values, indicating their potential biological significance, the details in Fig. 1A–B.

### MIR17HG -derived miRNAs

**Fig. 2:**
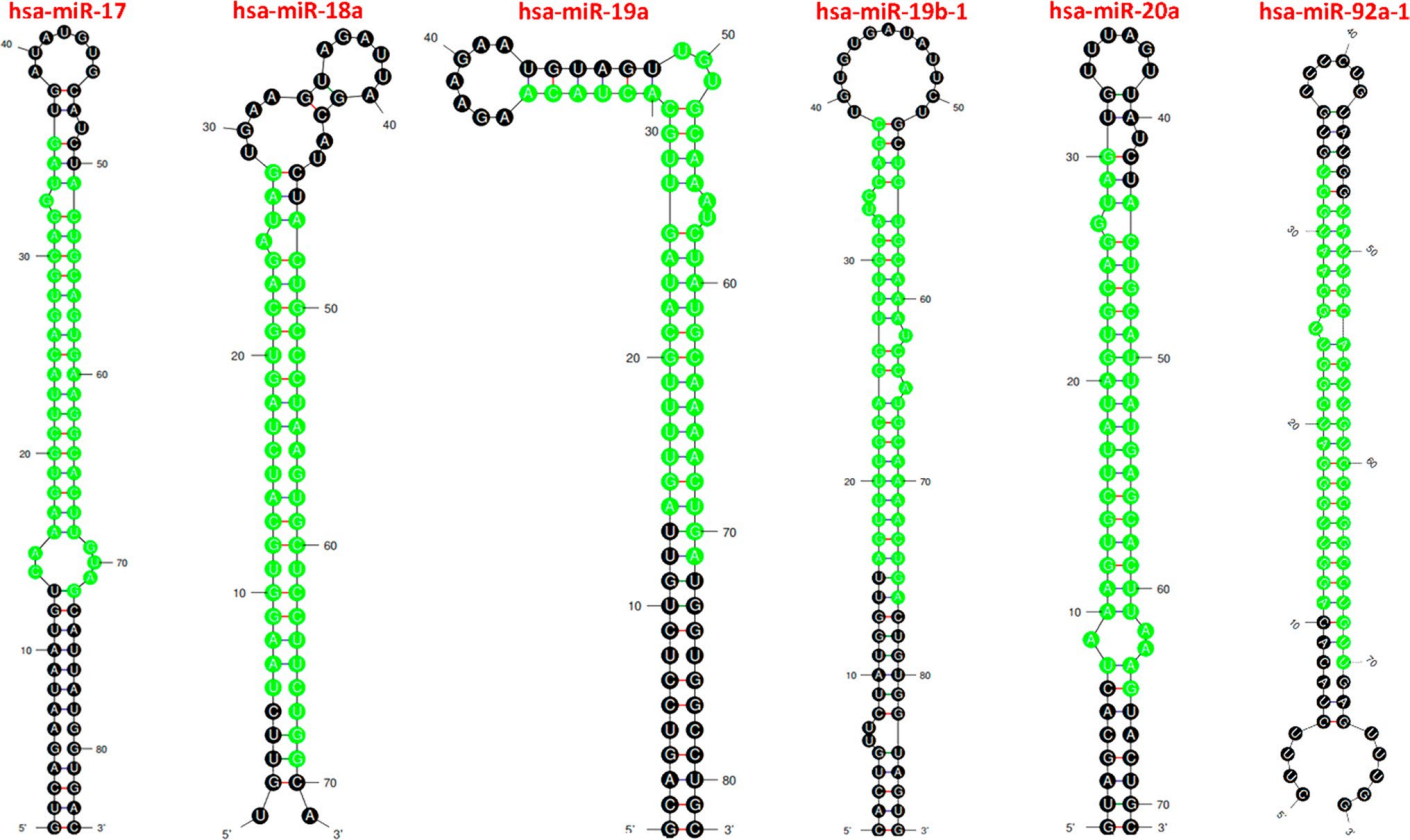
The optimized secondary structure of the MIR17HG-related miRNAs, which includes hsa-miR-17 (ΔG = − 34.30), hsa-miR-18a (ΔG = − 22.00), hsa-miR-19a (ΔG = − 39.10), hsa-miR-19b-1 (ΔG = − 38.40), hsa-miR-20a (ΔG =− 31.00), and hsa-miR-92a-1 (ΔG = − 35.70) stem-loop precursor miRNAs (pre-miRNAs). The green highlighting in the structure indicates the position where the mature miRNAs originate

We utilized the mfold web server (http://unafold.rna.albany.edu/?q=node/60) [19] to visualize the optimized secondary structures and the precise location of MIR17HG-related mature miRNAs (identified by green highlights) derived from their precursor (pre-miRNAs). This analysis allows us to obtain a clear and in-depth understanding of the miRNA’s location and structure (Fig. 2**)**. Table 1 presents comprehensive attributes of six pre-miRNAs that are linked with the miR-17-92a-1 cluster host gene, namely hsa-miR-17, hsa-miR-18a, hsa-miR-19a, hsa-miR-19b-1, hsa-miR-20a, and hsa-miR-92a-1.

**Table 1:** The fundamental details of six microRNAs that originate from the miR17HG

miRNAs	miRBase Accession Number	Minimum Free Energy (MFE) kCal/mol	Mature miRNA	Mature miRNA sequence
hsa-miR-17	MI0000071	− 34.30	hsa-miR-17-5p	CAAAGUGCUUACAGUGCAGGUAG
			hsa-miR-17-3p	ACUGCAGUGAAGGCACUUGUAG
hsa-miR-18a	MI0000072	− 22.00	hsa-miR-18a-5p	UAAGGUGCAUCUAGUGCAGAUAG
			hsa-miR-18a-3p	ACUGCCCUAAGUGCUCCUUCUGG
hsa-miR-19a	MI0000073	− 39.10	hsa-miR-19a-5p	AGUUUUGCAUAGUUGCACUACA
			hsa-miR-19a-3p	UGUGCAAAUCUAUGCAAAACUGA
hsa-miR-19b-1	MI0000074	− 38.40	hsa-miR-19b-1-5p	AGUUUUGCAGGUUUGCAUCCAGC
			hsa-miR-19b-1-3p	UGUGCAAAUCCAUGCAAAACUGA
hsa-miR-20a	MI0000076	− 31.00	hsa-miR-20a-5p	UAAAGUGCUUAUAGUGCAGGUAG
			hsa-miR-20a-3p	ACUGCAUUAUGAGCACUUAAAG
hsa-miR-92a-1	MI0000093	− 35.70	hsa-miR-92a-1-5p	AGGUUGGGAUCGGUUGCAAUGCU
			hsa-miR-92a-3p	UAUUGCACUUGUCCCGGCCUGU

### MIR17HG and miRNAs expression analysis

The clinical data of Colon Adenocarcinoma (COAD) were obtained from The cancer genome atlas (TCGA) database (https://portal.gdc.cancer.gov/repository) [20], specifically to analyze RNA-sequencing (RNA-seq) and microRNA-sequencing (miRNA-seq) data. The inclusion criteria were as follows: (1) verification of a histopathological diagnosis of COAD; (2) confirmation of the primary tumor site as the colon; (3) categorization of the disease type as adenoma or adenocarcinoma. Cases lacking complete demographic information, including gender and vital status, as well as those lacking comprehensive RNA-seq and miRNA-seq transcriptome profiling data, were systematically excluded. Ultimately, our study encompassed 380 COAD patients for RNA-seq data analysis and 369 COAD patients for miRNA-seq data analysis. The clinical characteristics of the patients are summarized in Table 2.

**Table 2:** Demographic data of COAD patients

	RNA-seq data analysis		miRNA-seq data analysis	
Characteristics	Number	Frequency (%)	Number	Frequency (%)
*Gender*				
Male	202	53.1	194	52.5
Female	178	46.9	175	47.5
Age (year) (mean (SD))	67 (12.7)		66.9 (12.8)	
< 69	189	49.7	184	49.8
≥ 69	191	50.3	185	50.2
*Vital status*				
Alive	301	79.2	290	78.5
Dead	79	20.8	79	21.5

The corresponding high-level data from RNA-Seq and miR-Seq were acquired using TCGA database. Raw read counts from RNA-Seq and miR-Seq datasets underwent normalization using the Trimmed Mean of M values (TMM) method. Differential expression analysis was conducted to identify differentially expressed long non-coding RNAs (DElncRNAs) and microRNAs (DEmiRNAs) between healthy solid tissues and primary malignancies. This analysis was executed using The “limma” package [21, 22] and Voom statistical analysis [23] within the R programming software (R-4.2.1, 64-bit, https://www.r-project.org/) [24], alongside RStudio Desktop version 2022.7.0.548 [25]. Subsequently, the miRNAmeConverter package [26] was employed to find the latest names of final DEmiRNAs from the miRbase version 22 database [27–32]. Furthermore, we employed GENT2, a platform designed for the exploration of gene expression patterns across normal and tumor tissues, to validate the DElncRNAs identified using Affymetrix microarray data from the GPL570 platform [33]. To validate the DEmiRNAs, we utilized microarray data from the GSE53592, GSE35834, and GSE18392 data series, sourced from the dbDEMC version 3.0 database, a comprehensive repository of differentially expressed miRNAs in human cancers [34]. Data filtering criteria for RNA/miRNA-seq and microarray data included a requirement for |log_2_ Fold Change (FC)|> 1, as well as a significance threshold based on *P*-value and adjusted *P*-value (false discovery rate (FDR)) < 0.01.

**Fig. 3:**
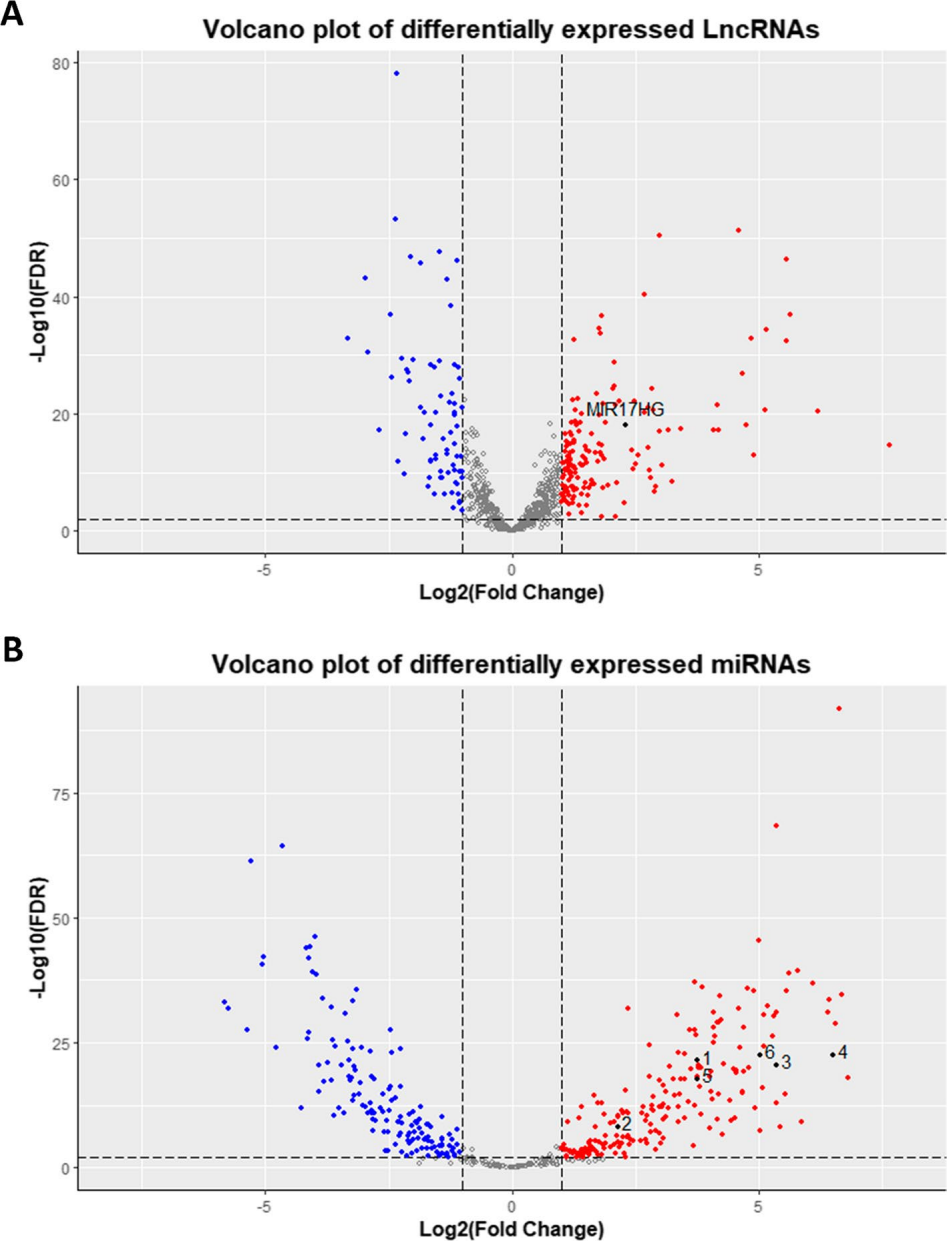
Volcano plots of the DELncRNAs and DEmiRNAs from RNA/miRNA-seq data. **A** The volcano plot depicts the DELncRNAs, with MIR17HG highlighted in black. **B** Volcano plot of DEmiRNAs denoting hsa-miR-17-5p, hsa-miR-17-3p, hsa-miR-18a-5p, hsa-miR-19a-3p, hsa-miR-19b-1-5p, and hsa-miR-20a-5p as data points 1 to 6, respectively. Over-expressed genes are represented in red, while under-expressed ones are displayed in Blue

The RNA-seq and microarray analysis results revealed that MIR17HG exhibits significant overexpression in tumor tissues compared to their normal (Table 3**, **Fig. 3A). Alternatively, while the expression profiles of hsa-miR-18a-3p, hsa-miR-20a-3p, and hsa-miR-92a-3p appeared inconsistent between miRNA-seq and microarray analyses, the other MIR17HG-derived miRNAs, including hsa-miR-17-5p, hsa-miR-17-3p, hsa-miR-18a-5p, hsa-miR-19a-3p, hsa-miR-19b-1-5p, and hsa-miR-20a-5p, collectively demonstrated an upregulation pattern in patients diagnosed with COAD (Table 4, Fig. 3B).

**Table 3:** The MIR17HG RNA-seq and microarray data analysis

	TCGA database		GENT2 database		Expression pattern
LncRNA	LogFC	FDR	LogFC	*P*-value	
MIR17HG	2.28	0.00***	1.047	0.00***	Up-regulation

*** < 0.001

**Table 4:** The MIR17HG-derived miRNAs miRNA-seq and microarray data analysis

	TCGA database		dbDEMC database			Expression Pattern
miRNA	LogFC	FDR	LogFC	Adjusted *P*-value	Data series	
hsa-miR-17-5p	3.75	0.00***	1.15	0.00***	GSE35834	Upregulation
hsa-miR-17-3p	2.13	0.00***	0.98	0.00***	GSE18392	Upregulation
hsa-miR-18a-5p	5.36	0.00***	1.13	0.003	GSE53592	Upregulation
hsa-miR-18a-3p	− 1.31	0.002	0.52	0.00***	GSE18392	Contradiction
hsa-miR-19a-5p	–	NS	–	NS	–	–
hsa-miR-19a-3p	6.51	0.00***	0.68	0.00***	GSE18392	Upregulation
hsa-miR-19b-1-5p*	3.75	0.00***	0.45	0.00***	GSE35834	Upregulation
hsa-miR-19b-1-3p	–	NS	–	NS	–	–
hsa-miR-20a-5p	5.03	0.00***	0.62	0.00***	GSE35834	Upregulation
hsa-miR-20a-3p	4.66	0.00***	–	NS	–	–
hsa-miR-92a-1-5p	–	NS	–	NS	–	–
hsa-miR-92a-3p	− 2.03	0.00***	0.20	0.00***	GSE18392	Contradiction

*hsa-miR-19b-1 in the GSE35834 was considered as the hsa-miR-19b-1-5p; ***: < 0.001; NS: not significant with *P*-value and adjusted *P*-value (false discovery rate (FDR)) threshold < 0.01^.^

The diagnostic potential of MIR17HG and its associated definitive upregulated miRNAs in COAD was assessed through receiver operating characteristic (ROC) analysis from RNA/miRNA-seq data, which yielded metrics including the area under the curve (AUC) and a 95 percent confidence interval (CI). These analyses were performed using GraphPad Prism 9.5.1, with statistical significance defined as a *P*-value < 0.01.

**Fig. 4:**
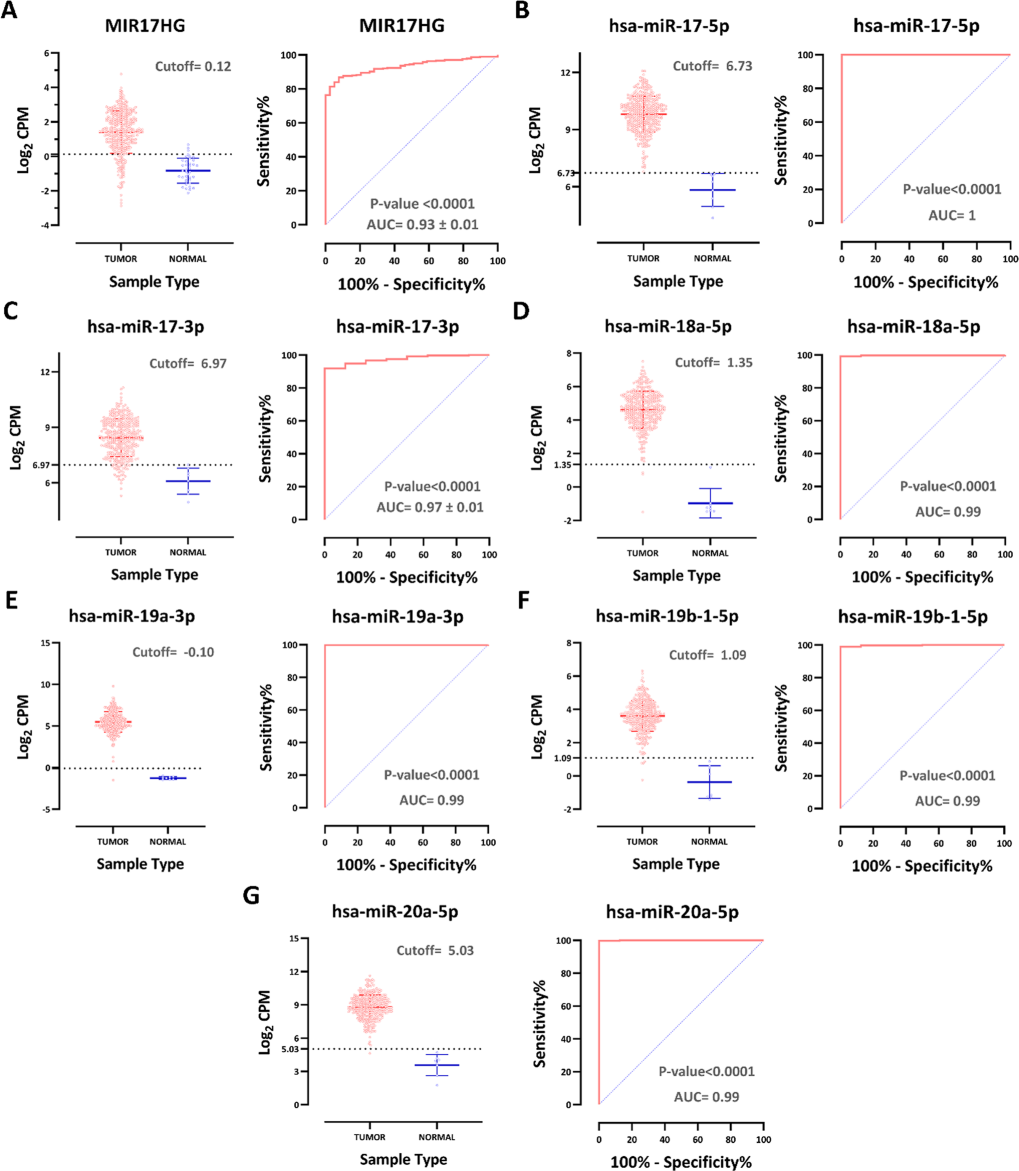
MIR17HG and its derived miRNAs diagnostic potential analysis. **A–G** Significantly increased expression of lncRNA MIR17HG and its derived miRNAs can distinguish tumor from normal tissue with different cutoff values and high specificity and sensitivity (*P*-value < 0.0001)

Our analysis revealed an impressive AUC value of 0.93 for MIR17HG (*P*-value < 0.0001, Fig. 4A). Furthermore, the AUC values of other microRNAs, including hsa-miR-17-5p, hsa-miR-17-3p, hsa-miR-18a-5p, hsa-miR-19a-3p, hsa-miR-19b-1-5p, and hsa-miR-20a-5p, were also evaluated. Remarkably, these microRNAs exhibited high AUC values of 1, 0.97, 0.99, 0.99, 0.99, and 0.99, respectively (*P*-values < 0.0001, Figs. 4B–G). This robust performance underscores the strong discriminatory power of MIR17HG and its associated miRNAs to distinguish COAD from normal tissues. Additionally, Fig. 4 serves as a pivotal resource by delineating the optimal cutoff values for distinguishing between tumor and normal tissue for each of these non-coding RNAs. Such delineation further enhances the chance of clinical utility of these ncRNAs by providing precise guidelines for their application in COAD. Further studies and validation are warranted to affirm their clinical utility.

### MIR17HG/miRNA/mRNA CeRNA axis

To elucidate the regulatory role of MIR17HG, we established a Competing Endogenous RNA (CeRNA) axis. The MIR17HG-associated miRNAs were identified by applying a stringent criterion, setting the CLIP region *P*-value to ≤ 0.01 utilizing the starBase v2.0 database (https://rnasysu.com/encori/index.php) [35, 36]. To harmonize the nomenclature with the latest miRNA standards, the miRNAs retrieved from starBase were subjected to the miRNAmeConverter package [26] aligning with miRbase version 22 [27–32]. Subsequently, we got an intersection between the down-regulated miRNAs from our TCGA miRNA-seq data analysis (LogFC < -1 and FDR < 0.01) and starBase miRNAs with the Venny 2.1 web tool (https://bioinfogp.cnb.csic.es/tools/venny/) [37]. Validated target mRNAs were identified using the multiMIR package, referencing data from miRecords, miRTarBase, and Tarbase databases [38]. Similarly, an intersection analysis was performed to align up-regulated mRNAs derived from our TCGA RNA-seq data analysis (with LogFC > 1 and FDR < 0.01) with the mRNAs from the multiMIR package, again utilizing the Venny 2.1 web tool. Data analysis was carried out using the R programming software (version R-4.2.1, 64-bit, https://www.r-project.org/) [24] in conjunction with RStudio Desktop (version 2022.7.0.548) [25]. Ultimately, the Cytoscape software (version 3.9.1) [39] was employed to construct the MIR17HG-miRNAs-mRNAs CeRNA axis, with the aid of the CytoHubba app [40] to identify central nodes within the network.

**Fig. 5:**
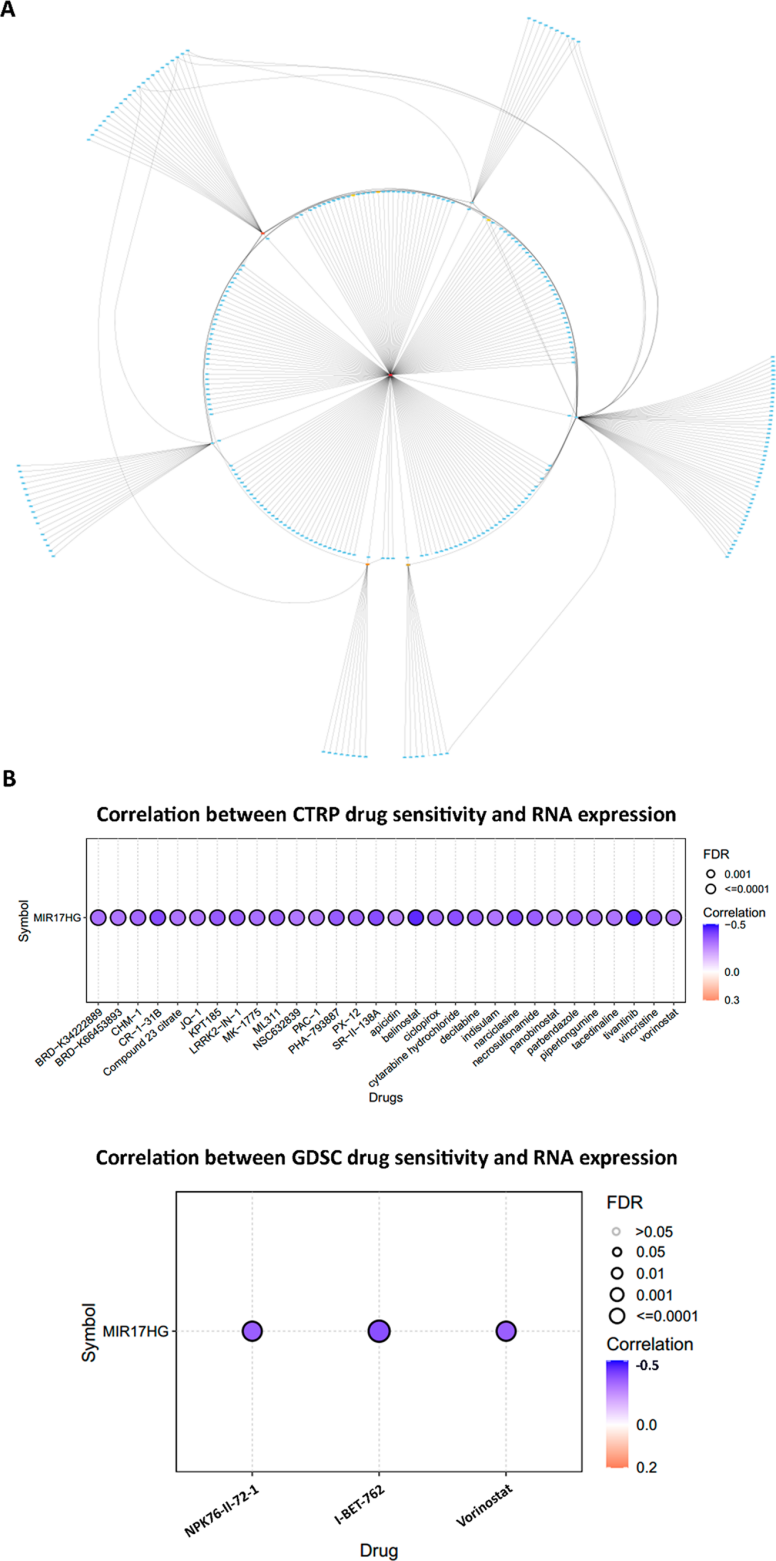
**A** The CeRNA network of the MIR17HG in COAD. **B** The drug sensitivity analysis for the MIR17HG gene using data from the GSCA database, showing the correlation between MIR17HG gene expression and drug sensitivity. A negative correlation indicates that elevated expression of the gene is associated with decreased drug sensitivity or potential resistance, while a positive correlation implies the opposite

Consequently, we have identified the top ten molecules according to the maximal clique centrality (MCC) ranking approach within the regulatory network of MIR17HG. Specifically, hsa-miR-214-3p, hsa-miR-140-3p, hsa-miR-501-3p, hsa-miR-1287-5p, hsa-miR-28-3p, hsa-miR-323a-3p, hsa-miR-664b-3p, CDCA4, NCAPG2, and MIR17HG were ranked with the highest MCC scores (Fig. 5A and Table 5). Collectively, these findings introduce the MIR17HG network as a central regulatory axis in the regulation of MIR17HG-mediated cellular processes in the context of COAD. However, it is crucial to emphasize that further experimental investigations are warranted to validate the intricate interactions among these nodes and MIR17HG, thereby enhancing our comprehension of the underlying molecular mechanisms governing COAD pathobiology.

**Table 5:** Top 10 nodes in MIR17HG-miRNAs-mRNAs network ranked by MCC method

Rank	Node Name	Score
1	hsa-miR-214-3p	175
2	hsa-miR-140-3p	72
3	hsa-miR-501-3p	34
4	hsa-miR-1287-5p	25
5	hsa-miR-28-3p	16
6	hsa-miR-323a-3p	12
7	hsa-miR-664b-3p	10
8	MIR17HG	7
9	CDCA4	4
9	NCAPG2	4

### MIR17HG drug sensitivity analysis

To investigate the potential utility of aberrant MIR17HG gene expression as a clinical response indicator and its suitability as a candidate predictive biomarker for drug screening, we conducted a drug sensitivity analysis utilizing the Gene Set Cancer Analysis (GSCA) database (http://bioinfo.life.hust.edu.cn/GSCA/#/) [41]. This database compiles the IC50 of several small molecules in human pan-cancer cell lines and its corresponding gene expression from two primary sources, the Cancer therapeutics response portal (CTRP) and Genomics of Drug Sensitivity in Cancer (GDSC) databases [42–47]. The analysis involves employing Pearson correlation to assess the relationship between gene expression and drug sensitivity, and the derived *P*-values are subjected to FDR adjustment. A negative correlation signifies that increased expression of the gene of interest corresponds to reduced drug sensitivity or potential resistance, and vice versa. While the outcomes from the GDSC database have suggested that cancer patients exhibiting high expression levels of the MIR17HG gene tend to display resistance to I-BET-762, NPK76-II-72–1, and Vorinostat, the CTRP results have further extended our understanding. The CTRP data have illuminated that elevated MIR17HG gene expression is likely associated with resistance to additional compounds, namely Belinostat, Tivantinib, and CR-1-31B. For a comprehensive overview of these correlations between MIR17HG expression and drug sensitivity, we refer to Fig. 5B, which visually encapsulates the intricate relationship between MIR17HG and drug responses. These findings collectively highlight the potential clinical significance of MIR17HG expression as an indicator of drug resistance in the context of cancer treatment. Given the absence of dedicated databases or datasets exclusively tailored for CRC, we have leveraged this comprehensive database to investigate the potential correlation between MIR17HG expression and drug sensitivity at the pan-cancer level. Nevertheless, it is essential to acknowledge the need for additional validation specifically within the context of CRC, as a means to address this particular limitation and enhance the robustness of our findings.

### MIR17HG co-expression analysis

The “Similar Gene Detection” module of GEPIA2, a gene expression profiling interactive analysis database (http://gepia2.cancer-pku.cn/#index) [48] was employed to identify a set of 500 genes exhibiting transcription profiles most closely resembling that of MIR17HG, drawing from the TCGA-COAD project. Only genes displaying a Pearson’s correlation coefficient ≥ 0.4 were considered for inclusion in this analysis. Subsequently, we assessed the expression patterns of the identified genes using our RNA-seq data and specifically selected those protein-coding genes that displayed significant differential expression (FDR < 0.01, LogFC > 1). Consequently, our focus was directed solely toward those protein-coding genes exhibiting a positive correlation with MIR17HG and concurrently displaying an upregulated expression pattern in COAD for subsequent analyses (63 protein-coding genes that are shown in supplementary material 1).

### MIR17HG gene set enrichment analysis

We conducted an enrichment analysis on the set of 63 mentioned mRNAs to elucidate the potential functional roles of protein-coding genes that exhibit co-expression with MIR17HG. This analysis encompassed gene ontology (GO) enrichment assessments across cellular component, molecular function, and biological process categories. Additionally, pathway enrichment analysis was performed, encompassing the Kyoto Encyclopedia of Genes and Genomes (KEGG), Wikipathway, and Reactome databases. The Enrichr tool (https://maayanlab.cloud/Enrichr/) was employed for these enrichment analyses, utilizing a significance threshold set at a *P*-value below 0.05 [49–51]. Furthermore, to visually represent the results of the GO and pathway enrichment analysis, dot plots were created. This was accomplished using the ggplot2 package [52] within the R programming software (version R-4.2.1, 64 bit, https://www.r-project.org/) [24] and RStudio Desktop (version 2022.7.0.548) [25]).

**Fig. 6:**
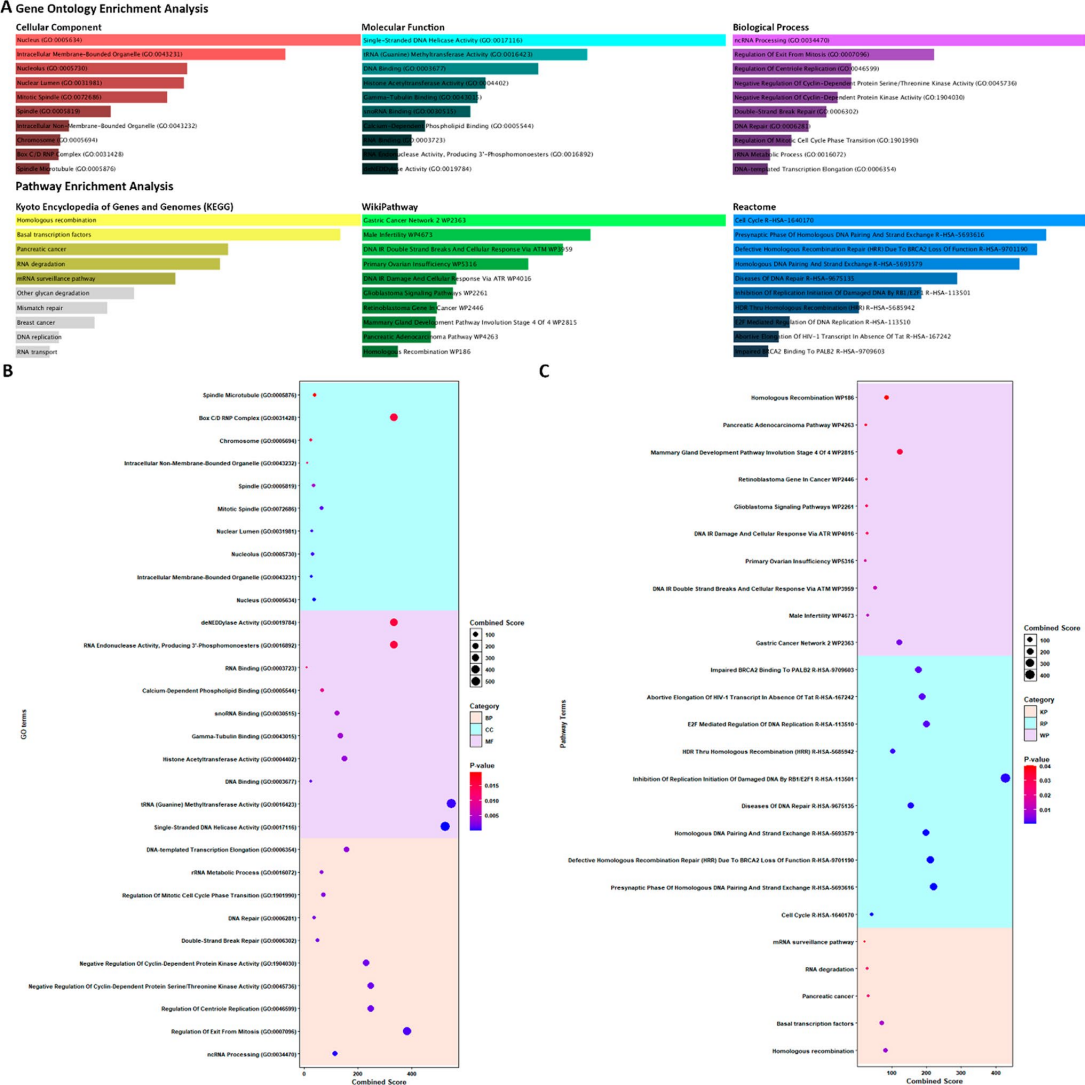
The MIR17HG-related gene set enrichment analysis. The GO enrichment analysis and pathway enrichment analysis from the Enrichr tool were visualized by bar plot **A** and dot plots **B, C,** highlighting statistically significant enrichments with a significance threshold set at *P*-value < 0.05

The findings from our analysis reveal significant involvement of the identified genes in a variety of crucial cellular processes. Notably, these genes play key roles in non-coding RNA (ncRNA) processing, the regulation of exit from the mitotic phase, and the control of centriole replication, highlighting their importance in fundamental biological mechanisms. Furthermore, our investigation unveiled that a substantial proportion of these genes exhibit enrichment in various molecular activities, including single-stranded DNA helicase activity, tRNA (Guanine) methyltransferase activity, as well as DNA and RNA binding. Additionally, some of these genes were associated with histone acetyltransferase activity, underscoring their potential influence on chromatin modification. In terms of cellular localization, our results indicate that these genes are predominantly situated within the cellular nucleus, with a noteworthy presence in the nucleolus. This subcellular distribution further emphasizes their essential roles in nuclear processes and gene regulation. Moreover, our analysis extends beyond functional annotations and encompasses insights into pathway involvement. We have identified prominent pathways based on comprehensive databases such as KEGG, Wikipathway, and Reactome. These pathways include Homologous recombination, basal transcription factors, DNA double-strand break repair and cellular responses via ATM (ataxia-telangiectasia mutated), and gastric cancer network pathways. These pathway associations shed light on the potential implications of the identified genes in various biological contexts (Fig. 6).

### Regulatory mechanisms of MIR17HG and its derived ncRNAs

**Fig. 7:**
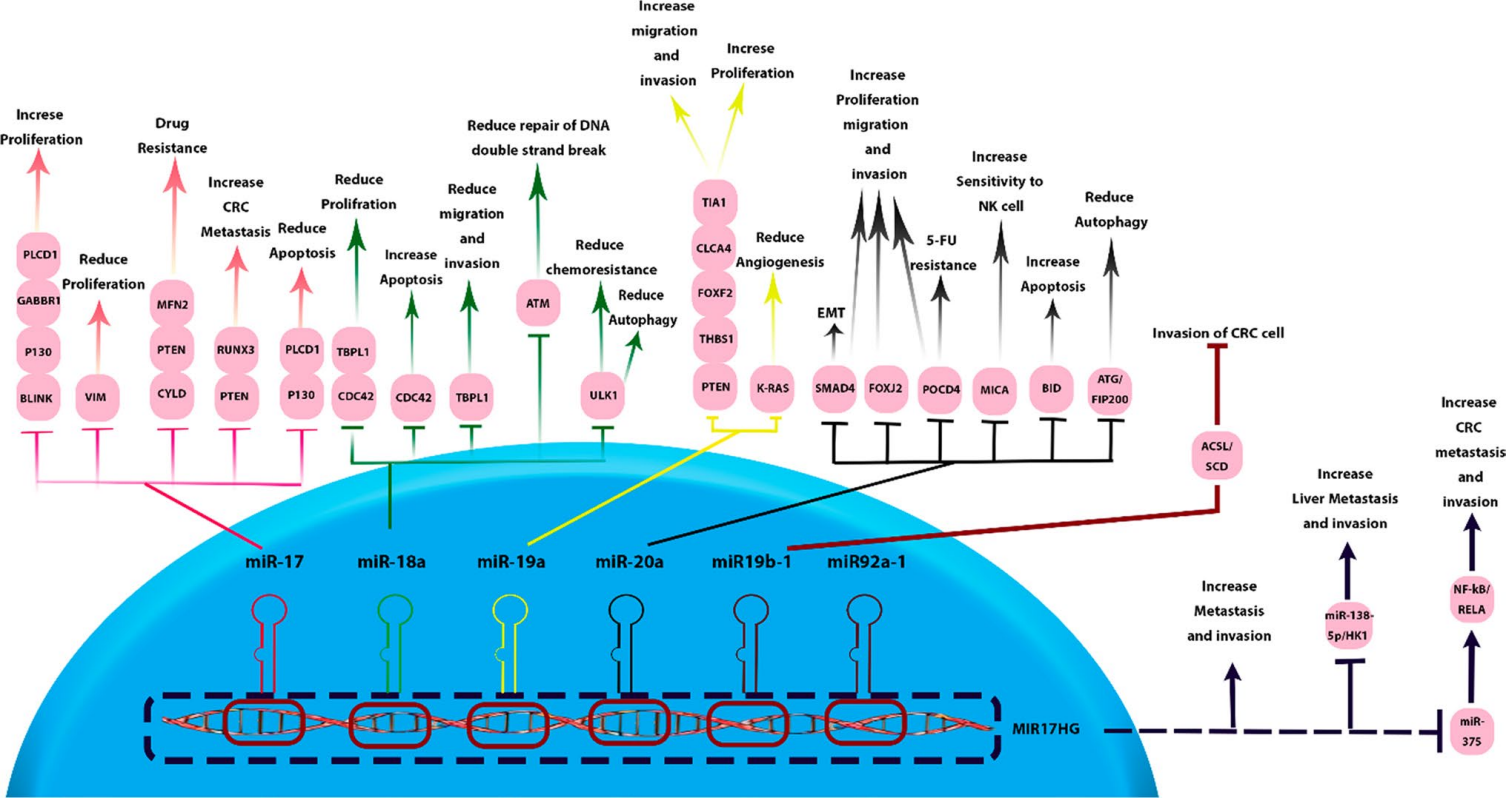
Regulatory mechanisms of MIR17HG and its associated miRNAs in CRC. MIR17HG and its derived miRNAs exert comprehensive regulatory mechanisms in CRC cells by targeting a diverse array of mRNAs. This cluster is recognized as a pivotal factor in the progression of CRC

The MIR17HG exerts multifaceted functions that are pivotal in normal developmental biology as well as in the pathogenesis of cancer. In the context of carcinogenesis, MIR17HG demonstrates a shift towards oncogenic activities. It actively promotes cellular proliferation, fosters a milieu conducive to angiogenesis, and inhibits cellular differentiation—all hallmarks of cancer progression. Furthermore, it contributes to the sustenance of cell viability, thereby potentially aiding in the persistent survival of malignant cells. These actions position MIR17HG as a crucial regulator within the cellular microenvironment [53]. Therefore, the specific mechanisms of MIR17HG and its associated miRNAs are delineated, elucidating their roles in the intricate network of gene regulation within oncogenic outcomes (summarized in Table 6 and Fig. 7).

**Table 6:** Regulatory Networks of MIR17HG and its associated ncRNAs in CRC oncogenesis

LncRNA/miRNA	Downstream target	Outcomes in CRC cells	Cell line	Refs.
MIR17HG	hsa-miR-375/NF-κB/RELA	Enhance invasion and metastasis of CRC cells	HCT116, SW620	[15]
	hsa-miR-138-5p/HK1	Enhance invasion of CRC cells / Increase liver metastasis	SW480, RKO	[54]
hsa-miR-17	MFN2	Resistance to 5-FU	HCT116	[55]
	BLINK	Enhance migration and invasion of CRC cells	SW620, HCT116	[15]
	PTEN	Metastatic behavior and multiple drug resistance	SW480, HCT116	[56]
	RUNX3	Enhance CRC metastasis	SW480, HCT116	[57]
	CYLD	Resistance to 5-FU	T84	[58]
	VIM	Decrease migration and invasion of CRC cells	LoVo, HT29	[59]
	P130	Increase proliferation and invasion / Decrease apoptosis	SW480, LoVo	[60]
	PLCD1	Reduce apoptosis, and increase invasion and migration of CRC cells	LoVo, HCT116	[61]
	GABBR1	Promote invasion and proliferation of CRC cells	HCT116, HT29	[62]
hsa-miR-19a	TIA1	Increase CRC cell invasion and proliferation	SW480	[63]
	CLCA4	Promote invasion, proliferation, and migration of CRC cells	CaCo2, SW480	[64]
	FOXF2	Increase CRC cell proliferation and migration	HCT116	[65]
	THBS1	Increase viability and migration of CRC cells	SW480	[66]
	PTEN	Enhance CRC cell migration and invasion	HCT116, SW480	[67]
	KRAS	Decrease angiogenesis of CRC cells	HCT116	[68]
hsa-miR-19b-1	ACSL/SCD	Impede invasion of CRC cells	SW620	[69]
hsa-miR-18a	CDC42	Reduce proliferation, and migration /Enhance apoptosis of CRC cells	HCT116, LIM1215	[70]
	TBPL1	Suppress growth, migration, and invasion of CRC cells	HCT116, SW620	[71]
	ULK1	Reduce autophagy activation/ Reduce Chemoresistance in CRC cells	HCT116	[72]
	ATM	Reduce DNA double-strand breaks repair	HCT116	[73]
hsa-miR-20a	BID	Reduce apoptosis of CRC cells	SW480	[74]
	MICA	Reduce CRC cell sensitivity to NK cells	SW480, HCT116	[75]
	FOXJ2	Increase proliferation, invasion, and migration of CRC cells	HCT116	[76]
	SMAD4	Induce EMT / increase proliferation and invasion of CRC cells	SW480	[77]
	POCD4	Increase CRC cells proliferation / Resistance to 5-FU	SW480, HCT116	[78]
	ATG5/FIP200	Inhibit the autophagic response initiated by hypoxia	LoVo, SW480	[79]

### MIR17HG

The long non-coding RNA MIR17HG enhances the metastatic and invasive capabilities of SW620 and HCT116 CRC cell lines. It functions as a CeRNA and modulates the expression of NF-κB/RELA by downregulation of miR-375 [15]. It enhances glycolysis activity in CRC cells through the regulation of the miR-138-5p/HK1 axis. Alternatively, overexpression of MIR17HG significantly increases the invasive potential of SW480 and RKO cell lines. Transplantation of these modified cell lines into nude mice increases the incidence of liver metastasis lesions [54].

### hsa-miR-17

**Fig. 8:**
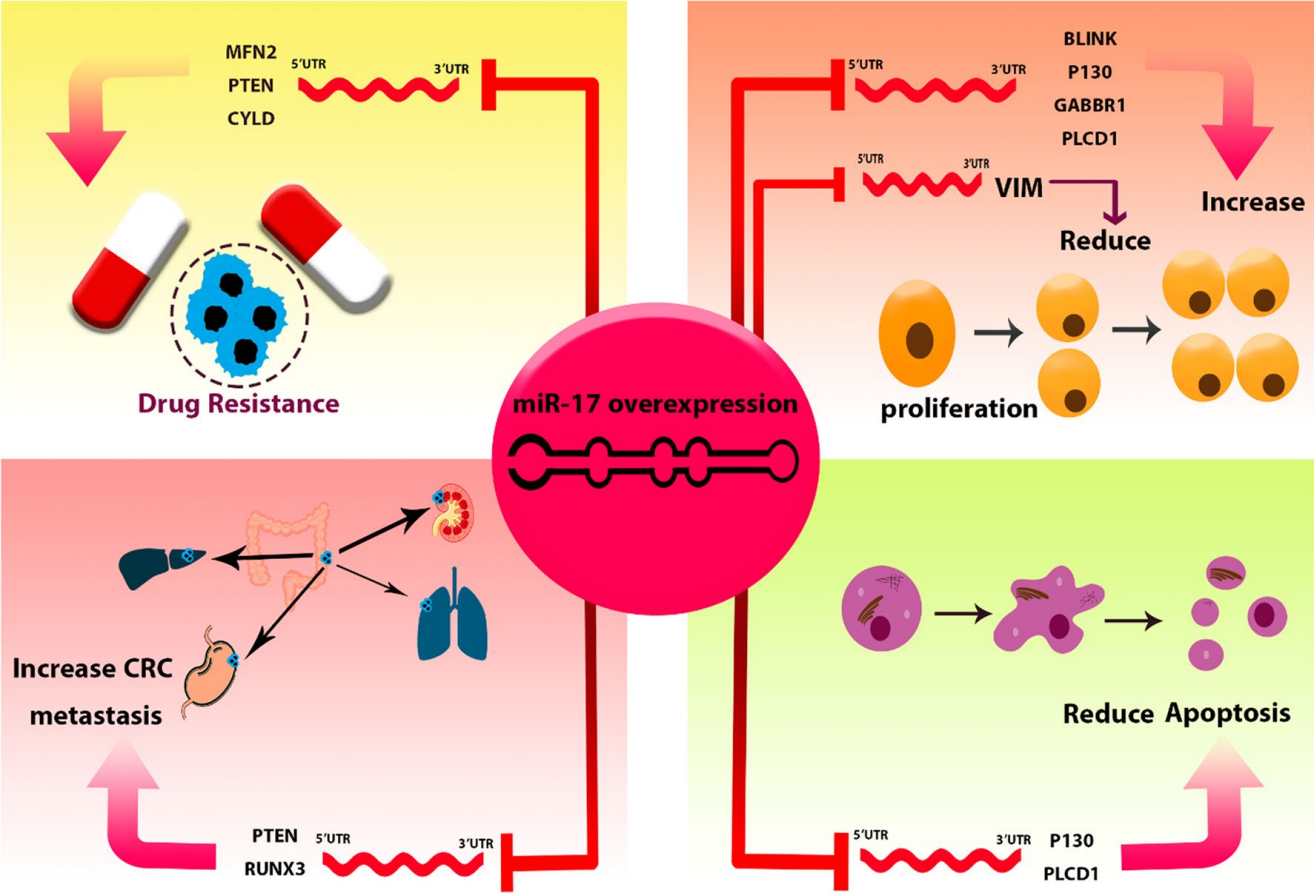
The oncogenic potential of hsa-miR-17 in CRC. miR-17 regulates various cellular processes, including cell proliferation and apoptosis. Its overexpression results in elevated drug resistance and metastasis in CRC cell lines

Studies conducted in the HCT116 cell line have provided insights into the regulatory role of miR-17-5p in colorectal cancer. This microRNA suppresses the expression of MFN2, leading to reduced mitochondrial fusion, increased mitochondrial fission, and enhanced mitophagy. These dynamic changes in mitochondrial behavior contribute to the development of resistance to 5-FU chemotherapy. Notably, these findings have been consistently replicated in mouse models, emphasizing their significance [55]. Furthermore, miR-17-5p directly targets BLINK mRNA and reduces its expression in SW620 and HCT116 cell lines, resulting in an enhanced capacity for invasion and migration in CRC cells [15]. Elevated expression of miR-17-5p in SW480 and COLO205 cell lines is associated with enhanced metastatic behavior and drug resistance. This outcome is attributed to the direct targeting of PTEN by miR-17-5p, with the down-regulation of PTEN playing a pivotal role in the promotion of metastasis and the acquisition of multiple drug resistance [56]. Additionally, using SW480 and HCT-116 cell lines, it was observed that miR-17-5p, by targeting RUNX3, activates the TGF-β signaling pathway and enhances colorectal cancer metastasis [57]. Pan et al*.* have indicated that miR-17 inhibits CYLD expression, thereby contributing to 5-FU resistance in the T84 cell line [58]. In primary colorectal cancer tissues, miR-17-5p is downregulated in the presence of metastasis compared to non-metastasis CRC, and it directly interacts with VIM mRNA. Increasing miR-17-5p expression reduces vimentin levels, thereby mitigating cell migration and invasion in LoVo and HT29 cells. In summary, miR-17-5p exerts a regulatory influence on vimentin expression, thereby impacting CRC metastasis [59]. Another study involving SW480 and LoVo cell lines revealed that miR-17-5p specifically targets P130, leading to the activation of the Wnt/β-catenin pathway and driving the progression of CRC by increasing the proliferation, invasion and migration, and decreasing apoptosis of CRC cells [60]. Alternatively, experiments carried out on CRC cell lines, specifically LoVo and HCT116, have revealed that the upregulation of miR-17-3p results in a decrease of apoptosis and a simultaneous enhancement of invasion, migration, and proliferation in CRC cells. These functional changes are attributed to the regulatory interaction between miR-17-3p and PLCD1 [61]. Zhang et al*.* demonstrated that exosomes derived from cancer-associated fibroblasts exhibit high expression of miR-17-5p compared to normal fibroblasts. miR-17 also exhibits a targeted binding to the 3'UTR of GABBR1 mRNA, resulting in heightened invasion and proliferation in HCT116 and HT-29 cell lines [62]. Generally, the oncogenic potential of hsa-miR-17 in CRC is summarized in Fig. 8.

### hsa-miR-19a / hsa-miR-19b-1

**Fig. 9:**
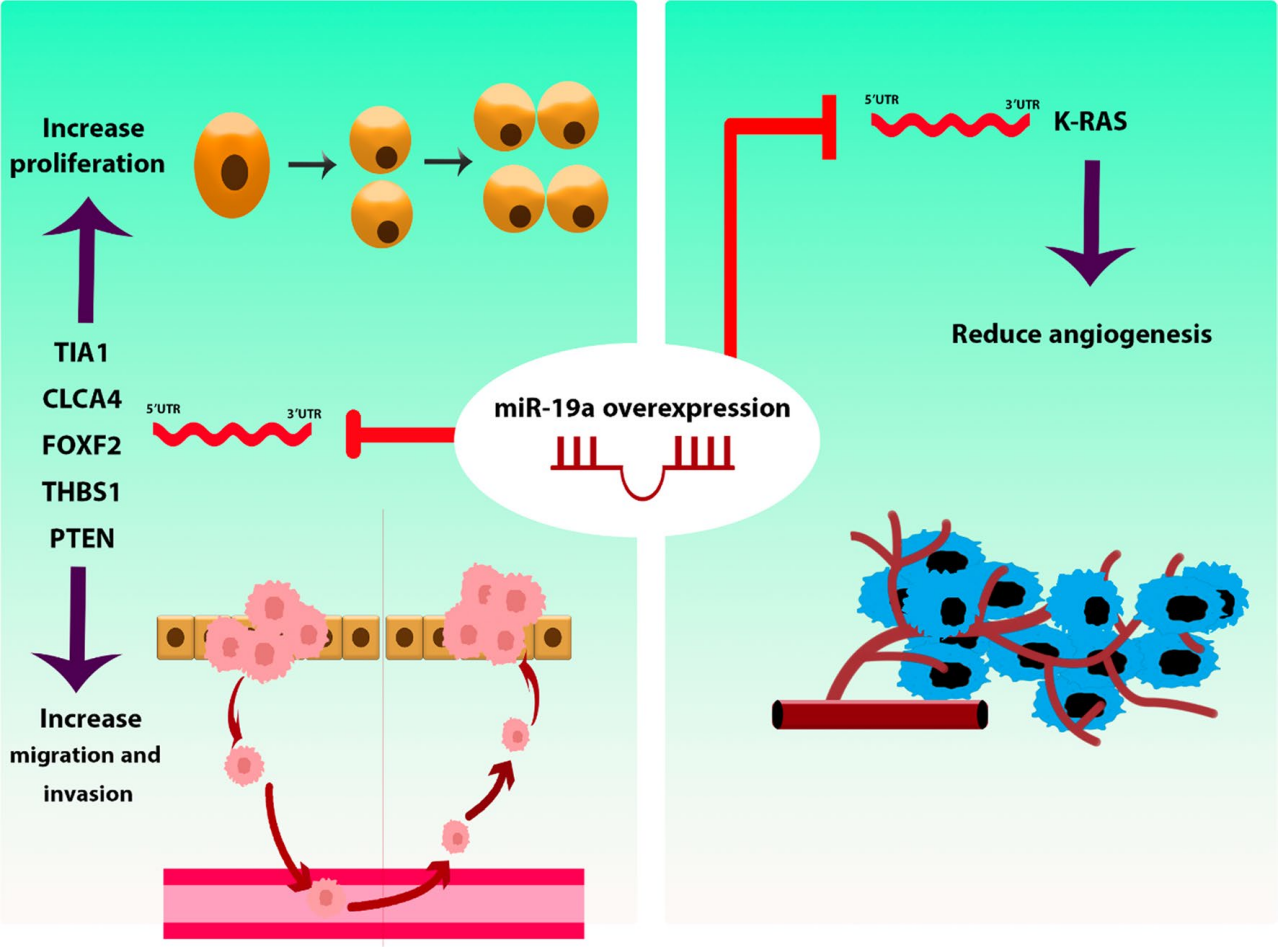
hsa-miR-19a regulatory mechanisms in CRC. miR-19a plays a pivotal role in controlling cell proliferation, invasion, and migration by targeting multiple mRNAs. Additionally, it regulates angiogenesis in CRC cells

miR-19a, via its interaction with TIA1, facilitates the promotion of migration and proliferation in SW480 CRC cells. Consistently, xenograft mouse models corroborated this observed effect [63]. The studies on CaCo2 and SW480 CRC cells established that miR-19a overexpression significantly increased the proliferation, invasion, and migration of CRC cells by under-expressing CLCA4. Furthermore, it was demonstrated that the miR-19a/CLCA4 axis plays a regulatory role in the PI3K/AKT pathway in these cell lines [64]. Alternatively, miR-19a-3p fosters proliferation and migration in HCT116 CRC cells by inhibiting the expression of FOXF2 [65]. Inhibiting the expression of miR-19a-3p can result in the elevation of the FOXF2-associated Wnt/β-catenin signaling pathway. This, in turn, impacts the epithelial-mesenchymal transition (EMT), cell proliferation, invasion, and cellular migration in both HT29 and HCT116 cells [80]. In their study, Yin and colleagues investigated the regulatory role of miR-19a in SW480 CRC cell lines. miR-19a was found to exert control over the viability, invasiveness, and migratory properties of CRC cells by directly interacting with THBS1. Notably, the application of a miR-19a inhibitor effectively mitigated the malignant characteristics of CRC cells, concomitantly leading to the down-regulation of matrix metallopeptidase 9 (MMP-9) and vascular endothelial growth factor C (VEGFC) expression [66]. In hypoxic conditions, miR-19a initiates the activation of the PI3K/AKT pathway, leading to an enhancement in cell migration and invasion within HCT116 and SW480 CRC cells. This functional modulation is primarily attributed to the targeting of PTEN mRNA by miR-19a [67]. In contrast to the prevailing consensus that regards miR-19a as an oncogenic factor, the findings by Chen et al*.* indicated that elevated miR-19a expression led to the inhibition of KRAS expression and subsequently curtailed angiogenesis in HCT116 cells. Importantly, this effect was consistently evident in a mouse model as well [68]. Figure 9 delineates a compendium of the regulatory mechanisms pertinent to hsa-miR-19a.

miR-19b-1 assumes a significant role as a central regulator of genes associated with lipid metabolism, specifically focusing on ACSL/SCD. miR-19b-1 directly oversees the pro-tumorigenic axis involving ACSL/SCD and exhibits the capability to impede invasion in SW620 and LoVo CRC cells. Importantly, it should be highlighted that diminished expression of miR-19b-1 is associated with a reduced survival rate among CRC patients, implying the potential involvement of ACSL/SCD in patient relapse [69].

### hsa-miR-18a

**Fig. 10:**
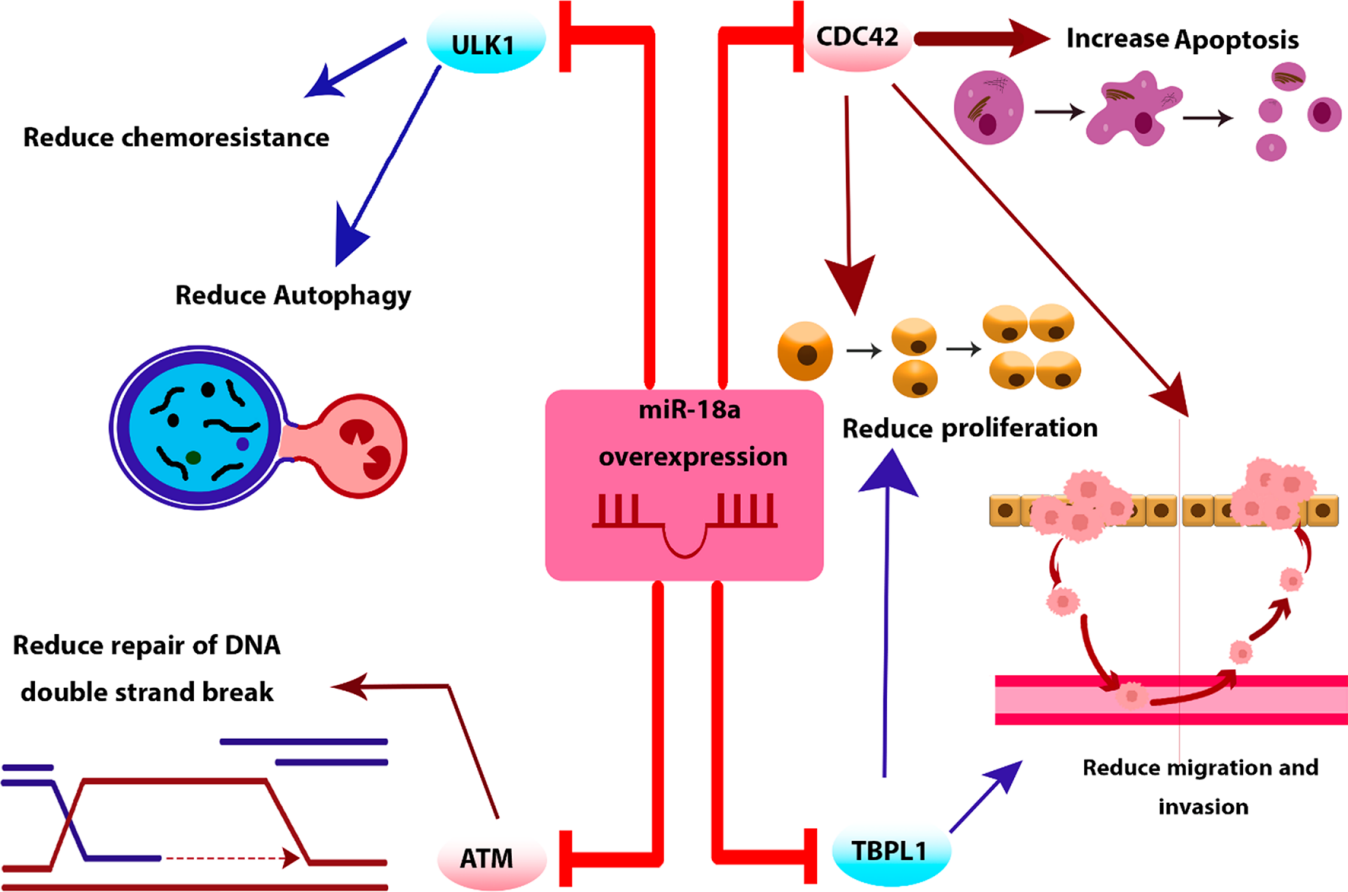
The various functions of Hsa-miR-18a in CRC. miR-18a regulates cell proliferation, invasion, migration, apoptosis, and autophagy. Additionally, it reduces chemoresistance and controls DNA double-stranded break repair in CRC cells

Mir-18a exhibits direct binding to the 3’ UTR of CDC42, a pivotal mediator within the PI3K pathway. Research conducted on HCT116 and LIM1215 CRC cell lines unveiled that miR-18a significantly reduces cell proliferation and migration while simultaneously enhancing apoptosis and the efficacy of pro-apoptotic agents [70]. Furthermore, it has been revealed that miR-18a exerts inhibitory effects on the growth, invasiveness, and migratory capabilities of HCT116 and SW620 colorectal cancer (CRC) cell lines. This regulatory influence is achieved through the specific targeting of TBPL1 by miR-18a [71]. Vu et al*.* brought to light the potential role of miR-18a in Fusobacterium nucleatum-mediated chemoresistance among CRC patients. Their research elucidated that miR-18a functions in the regulation of autophagy within HCT116 cells by suppressing ULK1 mRNA. Furthermore, their findings indicated that individuals with recurrent CRC exhibited elevated Fusobacterium nucleatum levels and diminished miR-18a expression when compared to patients who did not experience recurrence [72]. Moreover, in another study, it was demonstrated that miR-18a establishes a direct binding interaction with ATM mRNA. This interaction results in a reduction of DNA double-strand break repair within HCT116 CRC cells [73]. The different functions of hsa-miR-18a in CRC are delineated in Fig. 10.

### hsa-miR-20a

**Fig. 11:**
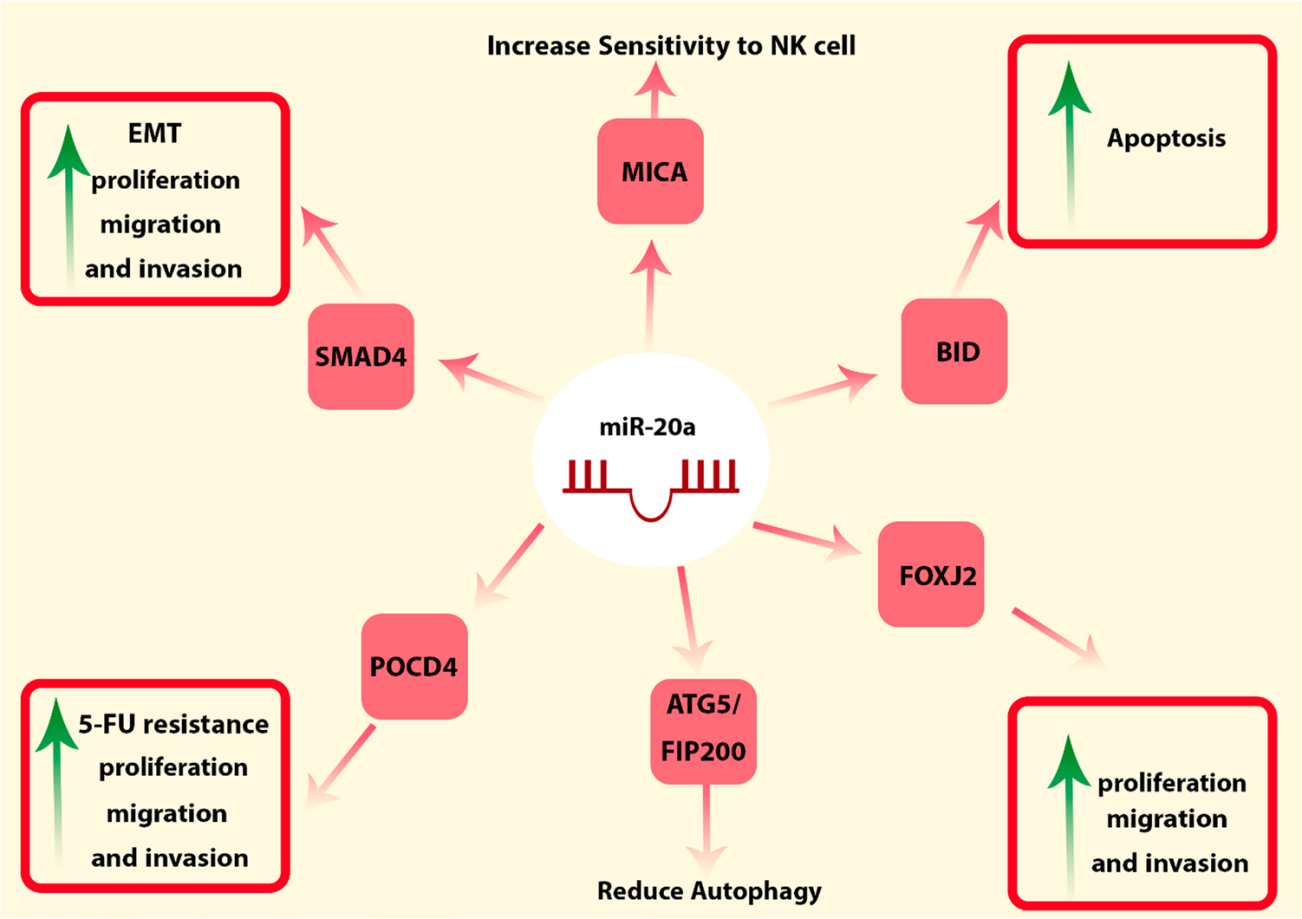
The regulatory roles of hsa-miR-20a in CRC. miR-20a controls cell proliferation, invasion, migration, autophagy, and apoptosis. This microRNA increases the CRC cells sensitivity to NK cells and also extends the resistance of these cells to 5-FU. Additionally, miR-20a facilitates EMT in CRC cells

Research conducted on SW480 cells revealed that miR-20a plays a role in the regulation of apoptosis by specifically targeting BID mRNA, a member of the pro-apoptotic gene family within BCL-2 [74]. miR-20a also influences the responsiveness of SW480 and HCT116 CRC cells to NK cells through its targeting of MICA [75]. miR-20a serves as a direct regulator of Foxj2, with experimental findings indicating that heightened miR-20a expression leads to a notable escalation in the proliferation, invasion, and migration of HCT116 CRC cells. This functional modulation is primarily attributed to the suppressive action of miR-20a on Foxj2 mRNA, underscoring its pivotal role in these cellular responses [76]. Zhang et al*.* indicated that miR-20a induced EMT and took part in the regulation of migration and invasion in SW480 cells, primarily by suppressing the expression of SMAD4 [77]. Moreover, It also directly binds to 3' UTR of POCD4 mRNA, resulting in the promotion of cell proliferation and resistance to 5-FU in SW480 and HCT116 CRC cell lines [78]. In another study, miR-20a displayed substantial downregulation compared to non-hypoxic conditions in colon cancer cells when subjected to hypoxic conditions. This reduction in miR-20a expression was found to impede the autophagic response triggered by hypoxia. Notably, miR-20a achieves this inhibitory effect by directly targeting several key regulators of autophagy, including ATG5 and FIP200, within LoVo and SW480 cell lines [79]. Figure 11 encapsulates the regulatory roles of miR-20a within CRC.

### Inflammatory bowel diseases and MIR17HG-derived ncRNAs

Inflammatory bowel diseases (IBDs) represent persistent intestinal disorders, commonly classified into two principal subtypes: Crohn’s disease (CD) and ulcerative colitis (UC) [81]. Recent investigations have brought to light the intricate role of MIR17HG-derived ncRNAs in the pathogenesis and control of essential molecular pathways associated with IBDs, several of which are elucidated below:

Wang et al*.* revealed that miR-20a-5p exhibited the most expression reduction in patients with CD compared to healthy controls. Their intervention, utilizing microspheres composed of poly (lactic-co-glycolic acid) and loaded with miR-20a-5p, led to remarkable enhancements in colitis, a decrease in mucosal inflammation, and an enhancement in the function of the epithelial barrier in mouse models of CD [82]. In a separate study, it was demonstrated that within the unaffected mucosal tissues of patients with CD, the expression of miR-18a and mir-20a was elevated in comparison to the control group [83]. Chen et al*.* identified a significant reduction in the expression of miR-19a and a remarkable increase in TNF-α within human colon tissues afflicted with UC [84]. However, findings from a study by Schaefer et al*.* indicated a significant elevation in miR-19a levels in both UC colon biopsies and blood samples compared to those from normal controls [85]. The serum levels of miR-19a-3p and miR-19b-3p were found to be lower in CD patients exhibiting a stricture phenotype compared to control CD patients. This analysis in patients with a 4-year follow-up period provided support for the hypothesis that diminished levels of miR-19a-3p and miR-19b-3p precede the development of strictures phenotype [86]. An examination conducted on IL-10 knockout mice revealed that the expression levels of ten miRNAs, including miR-19a, exhibited elevation in both the colon tissues and peripheral blood leukocytes compared to healthy control mice [87]. Additionally, there is supporting evidence indicating a significant upregulation of circulating miR-19b in individuals with IBD when compared to the control group [88].

Patients diagnosed with UC and CD encounter a substantial long-term concern, notably an elevated susceptibility to the development of CRC. In the context of CD, a noteworthy pattern emerges during the transition from non-neoplastic tissue to dysplasia, with miR-17 showing an upregulation. However, as the disease progression advances from dysplasia to full-blown cancer, a subsequent downregulation of miR-17 is observed [89]. This dynamic miR-17 expression profile signifies its intricate involvement in the multi-step process of CRC development within the context of CD, emphasizing the importance of further investigations into its regulatory mechanisms and potential implications for disease management.

## Conclusion and future prospect

The data presented in this study underscore the miR-17-92a-1 cluster host gene (MIR17HG) and its associated miRNAs upregulation in CRC, suggesting their significant involvement in the development and progression of CRC. They are implicated in critical oncogenic processes, including metastatic activity, apoptosis regulation, cell proliferation, and drug resistance. These findings shed light on the potential of MIR17HG and its associated miRNAs as therapeutic targets in CRC.

Future research should prioritize comprehensive investigations utilizing more human clinical samples to validate the oncogenic mechanisms of the miR-17-92a-1 cluster host gene and its associated miRNAs in CRC. Longitudinal studies would be particularly beneficial to trace miRNA expression over the cancer progression timeline. Additionally, functional studies aimed at exploring their background regulation by other molecular players in the CRC milieu could illuminate synergistic targets for intervention. Finally, developing advanced therapeutic strategies, including miRNA mimics or inhibitors, could pave the way for improved personalized treatment modalities for CRC patients.

## Supplementary Information

Below is the link to the electronic supplementary material.

Supplementary file 1 (XLSX 9 KB)

## Data Availability

The data used for this research are available from the corresponding author upon request.

## Abbreviations

CRC: Colorectal cancer
miRNA: MicroRNA
lncRNA: Long non-coding RNA
circRNA: Circular RNAs
MFE: Minimum free energy
COAD: Colon adenocarcinoma
TCGA: The cancer genome atlas
RNA-seq: RNA-sequencing
miRNA-seq: MicroRNA-sequencing
TMM: Mean of *M* values
DElncRNA: Differentially expressed long non-coding RNA
DEmiRNA: Differentially expressed microRNAs
FC: Fold change
FDR: False discovery rate
ROC: Receiver operating characteristic
AUC: Area under the curve
CI: Confidence interval
CeRNA: Competing endogenous RNA
MCC: Maximal clique centrality
KEGG: Kyoto Encyclopedia of Genes and Genomes
GSCA: Gene set cancer analysis
CTRP: Cancer therapeutics response portal
GDSC: Genomics of drug sensitivity in cancer
GO: Gene ontology
ncRNA: Non-coding RNA
ATM: Ataxia-telangiectasia mutated
MMP-9: Matrix metallopeptidase 9
VEGFC: Vascular endothelial growth factor C
IBD: Inflammatory bowel disease
CD: Crohn’s disease
UC: Ulcerative colitis
